# Glycinergic Modulation of Pain in Behavioral Animal Models

**DOI:** 10.3389/fphar.2022.860903

**Published:** 2022-05-25

**Authors:** Julian M. Peiser-Oliver, Sally Evans, David J. Adams, Macdonald J. Christie, Robert J. Vandenberg, Sarasa A. Mohammadi

**Affiliations:** ^1^ School of Medical Sciences, The University of Sydney, Sydney, NSW, Australia; ^2^ Illawarra Health and Medical Research Institute (IHMRI), University of Wollongong, Wollongong, NSW, Australia

**Keywords:** glycine receptor, glycine transporter, neuropathic pain, animal models, GlyT2 inhibitors, allosteric modulators

## Abstract

Animal models of human pain conditions allow for detailed interrogation of known and hypothesized mechanisms of pain physiology in awake, behaving organisms. The importance of the glycinergic system for pain modulation is well known; however, manipulation of this system to treat and alleviate pain has not yet reached the sophistication required for the clinic. Here, we review the current literature on what animal behavioral studies have allowed us to elucidate about glycinergic pain modulation, and the progress toward clinical treatments so far. First, we outline the animal pain models that have been used, such as nerve injury models for neuropathic pain, chemogenic pain models for acute and inflammatory pain, and other models that mimic painful human pathologies such as diabetic neuropathy. We then discuss the genetic approaches to animal models that have identified the crucial glycinergic machinery involved in neuropathic and inflammatory pain. Specifically, two glycine receptor (GlyR) subtypes, GlyRα1(β) and GlyRα3(β), and the two glycine transporters (GlyT), GlyT1 and GlyT2. Finally, we review the different pharmacological approaches to manipulating the glycinergic system for pain management in animal models, such as partial *vs*. full agonism, reversibility, and multi-target approaches. We discuss the benefits and pitfalls of using animal models in drug development broadly, as well as the progress of glycinergic treatments from preclinical to clinical trials.

## Introduction

**FIGURE 1:**
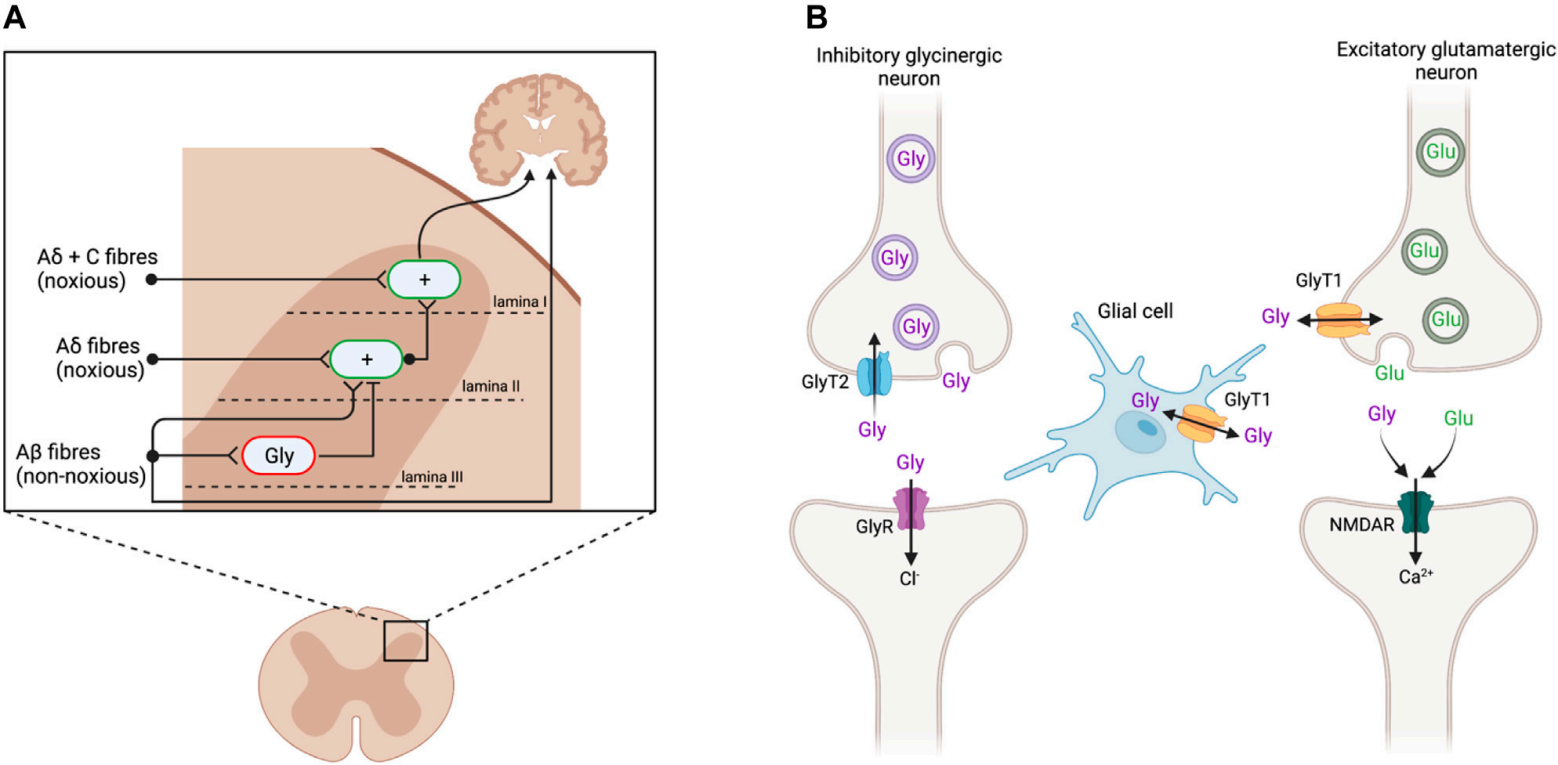
**(A)** Sensory input in the dorsal horn of the spinal cord. Aδ and C nerve fibers, transmitting noxious stimuli, synapse on excitatory interneurons (green; +) in the superficial laminae I and II. These signals are conveyed to the brain to elicit pain responses. Non-noxious stimuli transmitted *via* Aβ fibers innervate inhibitory glycinergic interneurons (red; Gly) in the deeper lamina III, inhibiting the activation of lamina II excitatory interneurons and preventing ascension of the pain signal. Following peripheral nerve injury, inhibitory glycinergic activity is reduced, resulting in a disinhibition of lamina II activation such that the non-noxious stimuli activate the nociceptive pathway. Adapted from Lu et al., (2013) and Vandenberg et al., (2014). **(B)** Glycinergic neurotransmission at inhibitory glycinergic and excitatory glutamatergic synapses. At inhibitory glycinergic synapses, glycine activates GlyRs, causing an influx of chloride ions to hyperpolarize the postsynaptic cell and inhibit the ascending pain signal. The concentration of glycine at GlyR (purple) is regulated by GlyT2 (blue), which transports excess glycine in the synaptic cleft back into the presynaptic cell for vesicular refilling. GlyT1 (yellow) regulates glycine and glutamate concentrations at NMDAR (green). Adapted from Supplisson and Roux (2002) and Vandenberg et al., (2014). Created with BioRender.

The spinal dorsal horn (DH) receives sensory information from primary afferent nerve fibers and relays signals to the brain (Figure 1A). This region has therefore been extensively studied to understand pain transmission and modulation, as well as targets for novel analgesics. Noxious stimuli are transmitted from the periphery to the brain *via* Aδ and C fibers that make their first synaptic connection at excitatory interneurons in the superficial DH laminae I and II. By contrast, Aβ fibers transmit signals from innocuous stimuli such as light touch to lamina III, where they also activate inhibitory glycinergic interneurons (Lu et al., 2013; Vandenberg et al., 2014). Activating these glycinergic interneurons inhibits the activity of excitatory neurons in lamina II, dampening the transmission of ascending pain signals. In chronic pain states that arise from damage to the somatosensory nervous system, inhibitory glycinergic activity is significantly reduced (Vandenberg et al., 2014; Imlach et al., 2016), resulting in disinhibition of the ascending pain pathway (Lu et al., 2013; Imam et al., 2020). Thus, non-noxious stimuli transmitted *via* Aβ fibers come to be perceived as painful. Animal models have proffered reliable routes for investigating these spinal changes through pharmacological and genetic studies.

### Glycine Transporters and Receptors as Drug Targets

Glycine acts as a neurotransmitter at both glycine (GlyRs) and *N*-methyl-D-aspartate (NMDAR) receptors. At inhibitory glycinergic synapses, glycine binds to GlyRs, resulting in hyperpolarization and subsequent inhibition of the postsynaptic cell (Figure 1B) (Lynch, 2004). At NMDAR, glycine acts as a co-agonist of glutamate, facilitating excitatory neurotransmission (Lynch, 2004; Vandenberg et al., 2014). The concentration of glycine at these synapses is regulated by its reuptake *via* glycine transporters (GlyT), of which there are two subtypes, GlyT1 and GlyT2. GlyT1 is more abundant and widely expressed by glial cells at inhibitory and excitatory synapses throughout the central nervous system (CNS), whereas GlyT2 expression is restricted to the presynaptic terminals of inhibitory glycinergic neurons in the spinal cord, brain stem, and cerebellum (Zafra et al., 1995; Zafra et al., 1997; Zeilhofer et al., 2018). GlyT1 and GlyT2 both remove glycine from the synaptic cleft to terminate signaling. GlyT2 specifically transports excess glycine in the synaptic cleft back into the presynaptic cell to reduce activation of GlyRs, while also ensuring enough glycine is available for presynaptic recycling and further inhibitory signaling (Supplisson and Roux, 2002). GlyT2 inhibitors are believed to increase glycine concentrations at GlyR to prolong inhibitory signaling and consequently produce analgesia (Vandenberg et al., 2014; Imam et al., 2020). By contrast, GlyT1 is present at both glycinergic and glutamatergic synapses, to regulate both excitatory signaling, *via* NMDAR, and inhibitory signaling, through GlyR (Supplisson and Roux, 2002; Vandenberg et al., 2014). The excitatory action of glycine at NMDAR may outcompete the inhibition at GlyR, resulting in net excitation at a given synapse, and so GlyT1 inhibitors may cause hypersensitivity rather than analgesia (Hermanns et al., 2008; Morita et al., 2008; Harvey and Yee, 2013).

GlyRs are ligand-gated ion channels with four *α* subunits and one *β* subunit (Lynch, 2004). They exist either as homomeric *α* pentamers or as heteromers with stoichiometries of 3α2β/2α3β/4α1β arranged to form a central chloride channel (Patrizio et al., 2017; Zhu and Gouaux, 2021). Under physiological conditions, glycine has its inhibitory action at GlyR by binding to the orthosteric site to cause an influx of chloride ions resulting in hyperpolarization. Direct spinal administration of the GlyR antagonist strychnine in mice results in disinhibition in the spinal cord, presenting as allodynia (Lu et al., 2013).

The β subunit of the GlyR is responsible for receptor clustering at the synapse, whereas the *α* subunit confers function and thus is the primary target for therapeutics (Patrizio et al., 2017). The GlyRα1 subunit is widely expressed throughout the CNS, whereas GlyRα3 expression is limited to the lamina II of the spinal DH (Malosio et al., 1991; Sato et al., 1991; Harvey et al., 2004).

GlyRα2 expression normally decreases after the postnatal period. However, in a rat neuropathic pain model, nerve injury results in reexpression of GlyRα2 at excitatory neurons in lamina II of the DH (Imlach et al., 2016). These findings align with increased DH expression of the Glra2 gene following spinal nerve ligation (Yu et al., 2019). The reason for this adaptation is not yet understood, thus further experimentation is required to elucidate the role of GlyRα2 in pain conditions. The restricted expression of GlyRα3 to the spinal DH and the unique reexpression of GlyRα2 only in neuropathic pain models make these two subunits excellent targets for analgesic drug development.

## Pain Models

**FIGURE 2:**
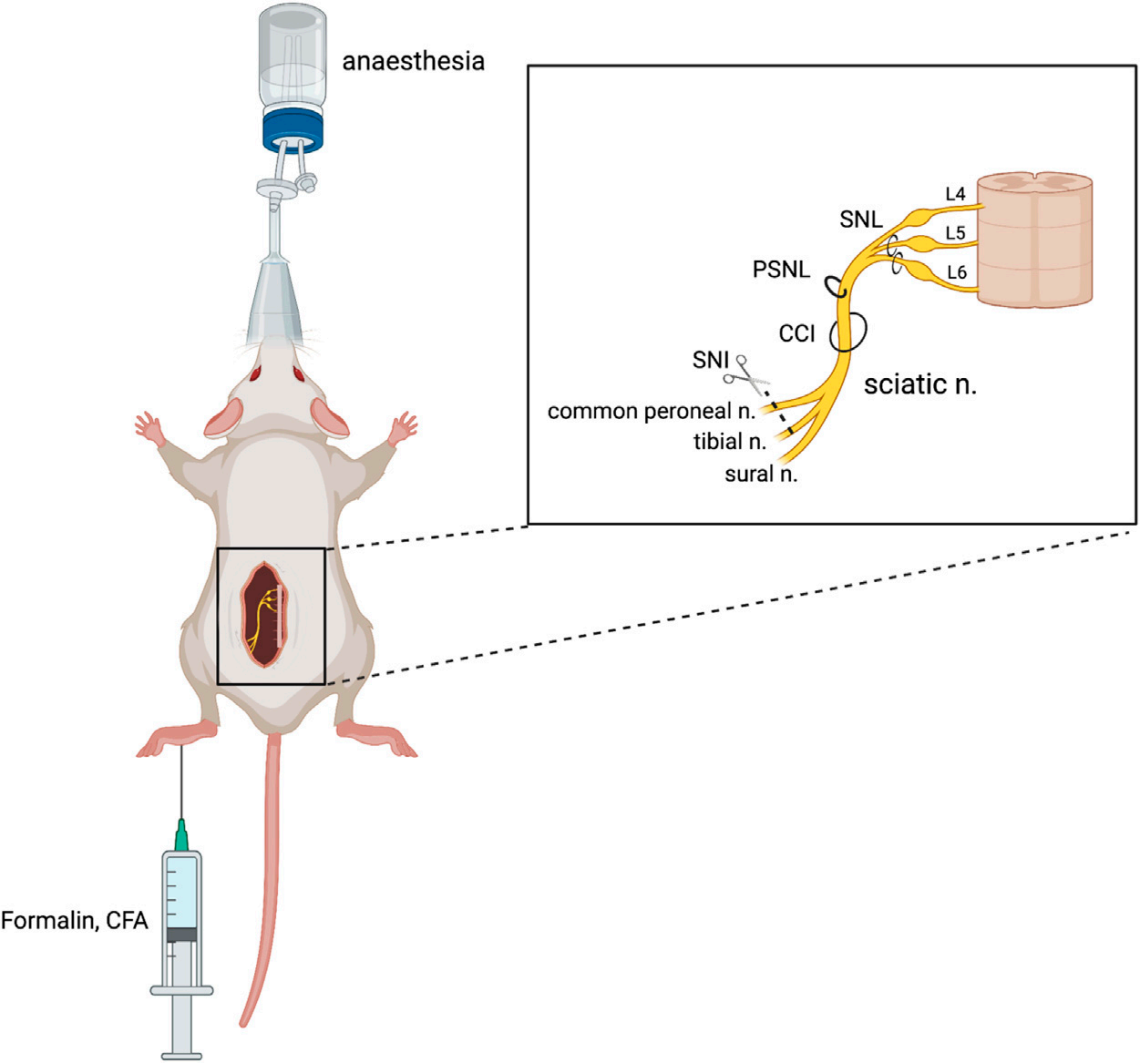
Animal models of chronic neuropathic pain. Neuropathic pain can be produced surgically *via* damage to a peripheral nerve, either through spinal nerve ligation (SNL), partial sciatic nerve ligation (PSNL), sciatic chronic constriction injury (CCI), or spared nerve injury (SNI). Chemical injection into the plantar surface of the hind paw can produce inflammatory (CFA) and neuropathic (formalin) pain models. Figure adapted from Bravo et al. (2020) and created with BioRender.com.

Various pain models have been successfully implemented in rodents to examine the glycinergic system. Here, we outline those animal models that have been used to date. Numerous variations of peripheral nerve damage, to spinal or sciatic nerves, have been used that induce neuropathy that is primarily neuropathic in etiology. Methods that use various chemical injections model acute chemical and neuroinflammatory pain as well as sub-chronic or chronic inflammatory pain. Disease models that mimic pain etiologies of human disease states have also been used and offer improved face and construct validity (Figure 2). Hypersensitivity is quantified based on predefined pain-like behaviors which are most often evoked rather than being spontaneous and are differentiated by the intensity of the stimulus evoking the behavior; allodynia presents where pain-like behaviors are evoked by a normally non-noxious stimulus (e.g., mechanical and thermal), while hyperalgesia presents as heightened sensitivity to a noxious stimulus (e.g., mechanical, thermal, and chemical).

### Surgical Models of Neuropathic Pain

Chronic constriction injury (CCI) of the sciatic nerve is a well-validated model of neuropathic pain which mimics peripheral nerve injury (Austin et al., 2012). This procedure was developed by Bennett and Xie (1988) in rats and involves the placement of several loose ligatures, traditionally of chromic catgut, around the sciatic nerve, causing inflammation and subsequent constriction of the nerve. The model was revised by Benbouzid et al. (2008) to replace the use of ligatures with cuffs. The cuff method has several benefits over the ligature method, namely, the fixed-diameter tubing that allows for consistent nerve compression across cohorts (Yalcin et al., 2014) and a relatively fast surgery time that minimizes any potential anaesthetic-induced side effects (Navarro et al., 2021). Through either method, CCI results in mechanical allodynia and thermal hyperalgesia, pain modalities that have been used to study glycinergic modulators (Lee et al., 1998; Hermanns et al., 2008; Armbruster et al., 2018). An increase in the mechanical and thermal withdrawal thresholds has been observed following the intraperitoneal (I.P.), subcutaneous (S.C.), and oral administration of GlyT1 inhibitor bitopertin (Armbruster et al., 2018) and intrathecal (I.T.) administration of ALX5407 (GlyT1 inhibitor) and ALX1393 (GlyT2 inhibitor) (Hermanns et al., 2008) in the CCI model.

Partial sciatic nerve ligation (PSNL) was first described by Seltzer et al. (1990) in rats and later adapted to mice (Malmberg and Basbaum, 1998). PSNL involves tight ligation of one-third to one-half of the sciatic nerve, denervating portions of the hind paw. PSNL has been used to study the physiological changes to glycine neurotransmission in neuropathic pain (Imlach et al., 2016) and potential pharmacological treatments, with inhibitors of both GlyT1 (ALX5407, ORG25935, and sarcosine) and GlyT2 (ORG25543, ALX1393, and oleoyl-D-lysine) reducing mechanical allodynia in rodents (Morita et al., 2008; Tanabe et al., 2008; Mostyn et al., 2019; Mohammadzadeh et al., 2021).

Spinal nerve ligation (SNL) is another model of neuropathic pain, developed by Kim and Chung (1992), where the L5 and L6 spinal nerves are tightly ligated and also produce significant mechanical allodynia. This procedure requires high technical skill to avoid damage to the L4 nerve which can abolish allodynia and cause motor deficits (Challa, 2015; Seto et al., 2021). This model has been used in rats to indicate glycinergic neurotransmission in the pathophysiology of pain, whereby oral or S.C. administration of the GlyT2 inhibitor opiranserin reduced mechanical allodynia in the von Frey assay (Pang et al., 2012).

The spared nerve injury (SNI) model is a variation of partial denervation developed by Decosterd and Woolf (2000) which allows mechanical testing of the paw adjacent to the injured areas. In the SNI model, two of the three sciatic nerve branches are transected, producing significant tactile and mechanical allodynia in mice (Bourquin et al., 2006; Huang et al., 2017). The resulting region of hypersensitivity granted by the spared sural nerve is on the lateral part of the hind paw, which poses limitations for testing (Decosterd and Woolf, 2000). This model was used by Bregman et al. (2017) to demonstrate that GlyRα1 and GlyRα3 potentiators can reverse tactile allodynia as measured by von Frey.

### Chemically Induced Pain Models

Other pain models include chemical injections that are localised to the tail, paw, or skin, or systemically administered via I.P. or S.C. injection. Complete Freund’s adjuvant (CFA) is a mineral oil containing heat-killed mycobacteria that are incapable of causing disease in animals (Navarro-Alvarez et al., 2018). Intraplantar injection of CFA evokes an inflammatory pain response at the site of injection, resulting in paw swelling and tactile allodynia in behavioral testing which is reduced following GlyT inhibitor administration (Ferreira et al., 2001; Kassuya et al., 2003; Morita et al., 2008; Chang et al., 2010; Abboud et al., 2021). Similar behavioural hypersensitivity is produced via intraplantar injection of PGE2 and Zymosan; both of these pain models have shown to be responsive to Glra3 point mutation (further discussed below; Werynska et al., 2021). These animal models have been used to mimic human conditions of chronic inflammatory pain.

The formalin test, first implemented by Dubuisson and Dennis (1977), results in biphasic pain, with an acute chemogenic pain phase followed by a long-lasting neuro-inflammatory phase thought to arise from central sensitization in the DH (McNamara et al., 2007). While the acute pain in phase I is adequately treated with NSAIDs and local anesthetics, phase II requires chronic pain treatments such as opioids and gabapentin, and it is in this phase where GlyT inhibitors are proposed to be active (McNamara et al., 2007; Tanabe et al., 2008). Chemically induced pain models are useful due to their efficiency. In the formalin model of acute pain, testing can commence within minutes of the injection time and does not require postsurgical recovery. However, this limited time span of testing may also confound results as the animals may experience elevated stress levels at the time of testing, exacerbating hyperalgesia (Jennings et al., 2014). The formalin test is commonly implemented and has been used to demonstrate the anti-allodynic effects of various GlyT2 compounds such as ORG25543, Compound 1, and opiranserin (Pang et al., 2012; Mingorance-Le Meur et al., 2013). Additionally, I.T. administration of GlyT1 inhibitor sarcosine inhibited the phase II nociceptive response following formalin injection (Tanabe et al., 2008).

Chemotherapy-induced peripheral neuropathy (CIPN) currently lacks effective treatments in humans. The painful neuropathy can be modeled in rodents via recurrent systemic injection of chemotherapy drugs. A GlyT2 inhibitor has recently been shown to be effective at reversing both mechanical allodynia and mechanical hyperalgesia in a rat CIPN model (Kuo et al., 2021).

### Disease-Induced Neuropathies

Animal models of disease-induced pain act as a proxy for studying the consequences of these complex human conditions. Diabetic rodents can be produced by administering streptozotocin, a glucose analog that selectively ablates pancreatic β islet cells, resulting in peripheral neuropathy, a complication of diabetes experienced by 50% of patients (Hicks and Selvin, 2019). The mechanism of painful diabetic neuropathy is complex, culminating in structural changes to the nerve fibers and altered synaptic transmission in the spinal DH. In rodent models, allodynia and hyperalgesia may develop (Morita et al., 2008; Ali et al., 2015). GlyT1 and GlyT2 inhibitors have been shown to increase the paw withdrawal threshold in von Frey testing of streptozotocin-induced diabetic mice, which suggests that the glycinergic system is involved in this pain model (Ali et al., 2015). Unlike surgical models, the diabetic model is limited by the additional symptoms of the induced pathology, such as hyperglycemia, weight fluctuation, and physical impairments (Hicks and Selvin, 2019).

Cancer pain most commonly develops as a result of nerve compression due to tumor growth (Caraceni and Portenoy, 1999). In particular, neuropathic cancer pain is most commonly associated with compression of or damage to the trigeminal nerve. A model of neuropathic cancer pain can be produced in rodents *via* injection of malignant cells, eventually leading to nerve compression or bone pain, which have been used to study glycinergic compounds in mice (Caraceni and Portenoy, 1999; Muralidharan et al., 2013). Motoyama et al. (2014) showed that at 11 days post-tumor implantation, the intravenous (I.V.), I.T., or oral administration of GlyT2 inhibitor ORG25543, as well as I.V. injection of GlyT2 inhibitor ALX1393 and GlyT1 inhibitor ORG25935, reduced allodynia, increased the paw withdrawal threshold and improved spontaneous pain behaviors (guarding and limb-use abnormality). Additionally, the authors showed that the simultaneous knockdown of spinal GlyT1 and GlyT2 had similar effects to the pharmacological interventions, which suggests that these compounds are acting on the spinal cord and have a similar mechanism of action as in other neuropathic pain models.

### General Considerations of Animal Models

The translation gap between preclinical and clinical efficacy has called into question the reliability of animal models of human pain conditions. A review by Herzberg and Bustamante (2021) reported failure rates of 90–95% in the clinical phase of drug development, and in 2010, it was revealed that the likelihood of an analgesic drug progressing beyond Phase I clinical trials was 10.7% (Hay et al., 2014; as cited in Herzberg and Bustamante, 2021). The poor translation from animal studies to humans in the clinic may be a culmination of limitations such as behavioral testing favoring the sensory over the emotional aspects of pain and insufficient diversity among study cohorts.

The validity and complexity of using animal pain models for preclinical translational research have recently been reviewed and discussed (Sadler et al., 2022). Here, we have reviewed those models that have been used in the context of glycine, which are limited to rodent models in the current literature.

Animal models of chronic pain are adept at producing quantifiable pain-related behaviors (e.g., limb withdrawal, spino-bulbo-spinal reflexes, vocalization, and licking of the hind paw) which have been crucial in understanding the pathophysiology of pain (Herzberg and Bustamante, 2021). However, there are concerns regarding the ability of these models to reflect the emotional facets of pain (King et al., 2009; Herzberg and Bustamante, 2021). To attain a more complete perspective of pain in animal studies, it has been suggested to analyze evoked pain outcomes, *via* mechanical or thermal stimulation, alongside non-evoked tests which better assess the quality of life (Mogil, 2009; Burma et al., 2017; Herzberg and Bustamante, 2021). For example, conditioned place preference experiments can determine if an analgesic drug is perceived as rewarding, indicating an overall improvement in the experience of the rodent (King et al., 2009). The grimace scale enables the study of spontaneous pain which experimenters can gauge by observing the facial language in rodents, resembling the approach to human pain treatment (Langford et al., 2010; Whittaker et al., 2021). Additionally, tests such as gait analyses and mechanical conflict-avoidance assays can be used to determine ongoing pain behaviors in a non-evoked manner (Harte et al., 2016; Deuis et al., 2017; Sadler et al., 2022). Together, these tests will better reflect a wider range of pain symptoms, which should improve the translatability of pain studies across species.

The homogeneity of test groups, while controlling for variability, likely also contributes to poor translation from animals to the clinic. A review by Mogil (2009) revealed that 79% of studies published in *Pain* between 1996 and 2005 only studied male animals, neglecting gendered differences in pain pathophysiology. Moreover, few studies have investigated the use of analgesics in multiple strains simultaneously. This is an issue highlighted in another review which identified that C57BL/6 and 129 mice, two of the most commonly used strains, exhibit significant phenotypic differences in nociceptive, hypersensitivity, and analgesic assays (Lariviere et al., 2001). Therefore, results derived from the testing of these strains may not apply to other strains, and *vice versa*. Future analgesic assays should therefore be performed in both sexes and a variety of strains to better inform success in the clinic.

## Genetic Models

Genetic manipulation of specific glycinergic transporters and receptors has provided evidence for their physiological roles in the normal and pain-state conditions.

Complete inactivation of GlyT1 (GlyT1^−/−^) in mice produces hyperglycine-induced sensorimotor deficits and severe respiratory depression, followed by death within 1 day of birth (Gomeza et al., 2003a; Tsai et al., 2004). Heterozygous knockout mice (GlyT1^+/−^) do not exhibit such deficits and appear normal but do develop electrophysiological changes with glycine saturation at NMDAR, particularly in the hippocampus (Gomeza et al., 2003a; Tsai et al., 2004; Martina et al., 2005). Cre recombinase–mediated inactivation of glial GlyT1 does not appear to affect adult mice, indicating a greater role of this transporter in neuronal development (Eulenburg et al., 2010).

GlyT2^−/−^ mice display behaviors phenotypic of hyperekplexia, a rare genetic disorder in humans affecting glycine neurotransmission, such as spasticity and tremor, inability to right from a supine position, and reduced motor coordination, with mortality in the second postnatal week (Gomeza et al., 2003b; Latal et al., 2010). The absence of GlyT2 prevents vesicular reuptake of glycine, severely diminishing the further release of glycine into the synapse and thus glycinergic neurotransmission. As with GlyT1^+/−^, GlyT2^+/−^ mice exhibit a normal behavioral phenotype. In mouse PSNL and bone cancer models, SiRNA knockdown, reducing expression by 75%, of either transporter had anti-allodynic effects (Morita et al., 2008; Motoyama et al., 2014). In both pain models, the reduced allodynia lasted 1–2 days longer in GlyT2 knockdown mice than in GlyT1 knockdown mice, a difference that the authors attributed to the widespread distribution of GlyT1.

Taking advantage of the localization of GlyT2 in the DH of the spinal cord, Foster et al. (2015) generated a transgenic GlyT2:Cre mouse line that allowed direct manipulation of glycinergic interneurons in lamina III. Ablation or silencing *via* diphtheria or tetanus toxins provoked spontaneous pain and increased mechanical allodynia and both hot and cold hyperalgesia. In a CCI model, exogenous activation of these glycinergic interneurons significantly reduced mechanical allodynia and hot and cold hyperalgesia (Foster et al., 2015).

Complete impairment of GlyRα1 by loss-of-function frame mutations causes severe motor deficits symptomatic of hyperekplexia as well as respiratory depression, followed by death within 3 weeks of birth (Buckwalter et al., 1994; Kling et al., 1997; Büsselberg et al., 2001). Mice with knock-in mutations of GlyRα1, reducing maximal glycine current by 30–60%, experienced no changes in motor coordination or thermal algesia when compared to the wild type (Findlay et al., 2003). As with complete GlyRα1 impairment, these mice exhibited seizures, an increased startle response, and limb clenching, followed by death within 3 weeks of birth.

Despite its apparent role in neuronal development, mice lacking GlyRα2 exhibit normal CNS morphology and no overt behavioral phenotype (Young-Pearse et al., 2006). Mice lacking GlyRα2 (Glra2^−/−^) demonstrated normal nociceptive behavior in models of acute pain and after peripheral nerve injury (Kallenborn-Gerhardt et al., 2012). However, mechanical hyperalgesia induced by peripheral injection of zymosan was significantly prolonged in Glra2^−/−^ mice when compared with their wild-type littermates.

GlyRα3 is the most extensively characterized receptor subunit in animal models. Glra3^−/−^ mice do not exhibit adverse phenotypic behavior, although a later study observed irregular respiration in mice lacking the α3-containing receptor (Harvey et al., 2004; Manzke et al., 2010). Pain behaviors also appear normal, with Glra3^−/−^ mice exhibiting no differences in mechanical allodynia and thermal sensitivities when compared to wild-type mice (Harvey et al., 2009). In a PSNL model, Glra3^−/−^ mice did not exhibit reductions in mechanical and thermal hypersensitivities when compared with their wild-type littermates. The lack of effect of α3 knockout in a neuropathic pain model is consistent with prior PSNL experiments showing reduced inhibitory glycinergic activity and a reversion to α2 subunit–containing receptors in lamina II of the DH (Imlach et al., 2016).

GlyRα3 has been demonstrated to be an important mediator of central sensitization in inflammatory pain. In the mouse CFA model, elevated COX2 led to the spinal release of PGE2, which inactivated GlyRα3 *via* phosphorylation. This GlyRα3-mediated inactivation of inhibitory neurons contributes to the central mechanisms of chronic inflammatory pain. In Glra3^−/−^ mice, CFA produced acute pain symptoms mediated by peripheral inflammatory mediators but without the central sensitization, and they exhibited quicker recovery than wild-type mice (Harvey et al., 2004; Harvey et al., 2009). More recently, a mouse line carrying a Glra3 point mutation that prevents PKA-dependent phosphorylation of the receptor exhibited a significant reduction in PGE2- and zymosan-induced hyperalgesia when compared to wild-type mice (Werynska et al., 2021).

Mouse models with GlyRβ loss-of-function mutations have been used as models of hyperekplexia (Kingsmore et al., 1994; Becker et al., 2000; Harsing et al., 2006). The mutation caused a significant reduction in postsynaptic GlyR, eliciting behaviors seen in GlyRα1^−/−^ mice.

## Pharmacological Approaches

Compounds that potentiate GlyRs or inhibit GlyTs are expected to offer therapeutic benefits by increasing the affinity of glycine for GlyR or by elevating synaptic glycine concentrations. Thus, the inhibitory tone that is lost in the DH in chronic pain states may be restored (Huang et al., 2017). Screening novel compounds in animal models has contributed to the understanding of the role of glycine in pain and offered preclinical evidence of potential clinical success.

### GlyT1 Inhibitors

GlyT1 inhibitors were initially developed as antipsychotics to treat schizophrenia. These compounds function by increasing glycine concentrations around NMDAR to restore receptor functionality (Bergeron et al., 1998). It was later considered that inhibiting GlyT1 could increase glycinergic neurotransmission and produce analgesia. The main classes of GlyT1 inhibitors are sarcosine, an endogenous and competitive substrate for GlyT1, and its derivatives as described in Table 1 (Mezler et al., 2008).

**TABLE 1:** Experiments investigating known GlyT1 inhibitors. I.T., intrathecal; I.V., intravenous; S.C., subcutaneous; I.P., intraperitoneal; ROA., route of administration.

Compound	References	Model	Animal	Dose	ROA	End points
Sarcosine	Morita et al. (2008)	CFA	Mice	20 ng	I.T.	Reduced mechanical allodynia
		Diabetic		20 ng	I.T.	
				≤0.3 mg/kg	I.V.	
						Delayed reduction in mechanical allodynia
		PSNL				
	Tanabe et al. (2008)			10, 30 µg	I.T.	Reduced thermal, mechanical hypersensitivity
		Diabetic				Reduced mechanical hypersensitivity
		Formalin				Inhibited second phase nociception
ALX5407	Tanabe et al. (2008)	PSNL	Mice	0.03, 0.1 µg	I.T.	Reduced mechanical allodynia
		Diabetic				
		Formalin				Reduced formalin-induced pain
	Hermanns et al. (2008)	CCI	Rats	10, 50, 100 µg		Reduced allodynia at low and high doses, not at medium dose
	Barthel et al. (2014)			≥0.2 μg/kg	S.C. osmotic infusion	Reduced mechanical allodynia
	Mohammadzadeh et al. (2021)	PSNL		4 mg/kg	S.C.	
ORG25935	Morita et al. (2008)	CFA	Mice	300 ng	I.T.	Delayed reduction in mechanical allodynia
		PSNL		0.3 mg/kg	I.V.	
	Motoyama et al. (2014)	Bone cancer				Multiday allodynia reduction
N-ethylglycine	Werdehausen et al. (2015)	CFA	Mice	200 mg/kg	S.C.	Reduced mechanical hyperalgesia
		CCI				Reduced mechanical allodynia
Bitopertin	Armbruster et al. (2018)	CCI	Rats and mice	≤10 mg/kg	I.P. Oral S.C.	Reduced mechanical allodynia and thermal hyperalgesia
		Carrageenan inflammatory				

I.T. and I.V. administration of sarcosine has been shown to reduce nociceptive behaviors in PSNL, CFA, formalin, and diabetic neuropathy models in mice (Morita et al., 2008; Tanabe et al., 2008). In mouse models of herpetic and postherpetic neuralgia, I.T. sarcosine produced no analgesic effects, which may be due to the downregulation of spinal GlyT1 in these models (Nishikawa et al., 2010). The anti-allodynic effects of sarcosine in PSNL mice appeared with a 1- to 2-hr delay. This delay was also observed following I.V. injection of sarcosine-derived reversible and noncompetitive GlyT1 inhibitor, cis-N-methyl-N-(6-methoxy-1-phenyl-1,2,3,4-tetrahydronaphthalen-2-ylmethyl)amino-methylcarboxylic acid hydrochloride (ORG25935) (Morita et al., 2008; Lidö et al., 2017). By antagonizing the glycine binding site on NMDAR, the application of both sarcosine and ORG25935 produced a rapid onset of analgesia, confirming that the time lag was due to NMDAR activation (Morita et al., 2008). The delays seen in PSNL but not in other animal models may be due to the reexpression of the less glycine-sensitive GlyRα2, with reduced signaling unable to overcome NMDAR activation due to spillover (Imlach et al., 2016).

Spinal and I.V. administration of ORG25935 reduced allodynia in mouse diabetic neuropathic and bone cancer pain models and, to a lesser extent, in a CFA mouse model (Morita et al., 2008; Motoyama et al., 2014).

A lipid compound with a sarcosine headgroup, N-[3-(4′-fluorophenyl)-3-(4′-phenylphenoxy)propyl]sarcosine (ALX5407), was developed that binds irreversibly and noncompetitively to GlyT1s (Atkinson et al., 2001; Aubrey and Vandenberg, 2001). I.T. administration of ALX5407 reduced mechanical allodynia in PSNL and diabetic mouse models of neuropathic pain, as well as formalin-induced pain (Tanabe et al., 2008). In a rat CCI model, the spinal administration of ALX5407 produced anti-allodynic effects at high (100 μg) and low (10 μg) doses, with no significant effects at a medium dose (50 μg) (Hermanns et al., 2008). The loss of anti-allodynia with 50 μg was thought to be due to the synaptic spillover of glycine to nearby NMDAR, promoting excitation, although this does not explain anti-allodynia at higher doses. Several studies that examined ALX5407 as a treatment for schizophrenia observed respiratory depression and severe motor dysfunction following oral and I.P. administration in both rats and mice (Harsing et al., 2006; Perry et al., 2008). By binding irreversibly to GlyT1, ALX5407 administration can overstimulate both GlyR and NMDAR, producing side effects that mimic the GlyT1^−/−^ phenotype.

Two non-sarcosine-derived GlyT1 inhibitors, RG1678 (bitopertin) and N-ethylglycine, have also shown promising results *in vivo*. Bitopertin, a noncompetitive GlyT1 inhibitor, reduced mechanical allodynia and thermal hyperalgesia in a dose-dependent manner in CCI and carrageenan-induced chronic inflammatory murine models (Armbruster et al., 2018). I.P., oral, and S.C. administration of bitopertin produced a profound analgesic effect at low doses (2 mg/kg), comparable to 300 mg/kg gabapentin, with no observed side effects. Bitopertin reached clinical trials as a treatment for schizophrenia, failing at phase III testing, but it may have potential as an analgesic. N-ethylglycine is a lidocaine metabolite that selectively inhibits GlyT1 (Werdehausen et al., 2012). In a CFA mouse model, S.C. N-ethylglycine reduced mechanical hyperalgesia in a dose-dependent manner (Werdehausen et al., 2015). A single dose administered S.C. also reduced mechanical allodynia in a CCI mouse model.

The observation that the analgesic properties of GlyT1 inhibitors such as sarcosine, ORG25935, and ALX5407 are decreased or even counteracted by the stimulation of NMDAR in the spinal cord and higher brain regions hinders the development of GlyT1 as an approach to restoring glycinergic signaling in pain states.

### GlyT2 Inhibitors

GlyT2 inhibitors, described in Table 2, have been developed to modulate glycinergic neurotransmission, restoring the balance of synaptic and presynaptic glycine in chronic pain. O-[(2-benzyloxyphenyl-3-flurophenyl)methyl]-L-serine (ALX1393) is considered to be a selective GlyT2 inhibitor, reducing allodynia in CCI rat models *via* central administration (Hermanns et al., 2008; Barthel et al., 2014; Takahashi et al., 2015). In PSNL, formalin, and bone cancer pain models, ALX1393 reduced allodynia and hyperalgesia in wild-type mice, but this analgesia was lost in GlyRα3^−/−^ mice (Morita et al., 2008; Mingorance-Le Meur et al., 2013; Motoyama et al., 2014). At high doses, ALX1393 loses GlyT2 selectivity, inhibiting GlyT1 and activating nearby NMDAR, causing respiratory and motor side effects (Hermanns et al., 2008; Mingorance-Le Meur et al., 2013).

**TABLE 2:** Experiments investigating known GlyT2 inhibitors. I.C.V., intracerebroventricular.

Compound	References	Model	Animal	Dose	ROA	End points
ALX1393	Hermanns et al. (2008)	CCI	Rats	100 µg	I.T.	Reduced allodynia. Severe resp. depression
	Barthel et al. (2014)			Up to 100 μg/kg/day	S.C. osmotic infusion	Reduced mechanical allodynia, thermal hyperalgesia
	Takahashi et al. (2015)			100 µg	I.C.V.	Reduced allodynia, hyperalgesia
	Morita et al. (2008)	PSNL GlyRα3^−/−^	Mice	0.01 mg/kg	I.V. I.T.	Reduced allodynia, not in GlyRα3^−/−^ KO
	Motoyama et al. (2014)	Bone cancer			I.V. Oral	Reduced hyperalgesia
ORG25543	Mingorance-Le Meur et al. (2013)	Formalin	Mice	0.06–20 mg/kg	I.V.	Reduced mechanical allodynia. Seizures/death at higher doses.
	Morita et al. (2008)	PSNL GlyRα3^−/−^		0.3 mg/kg	I.V. I.T.	Reduced mechanical allodynia, not in GlyRα3^−/−^ KO
	Motoyama et al. (2014)	Bone cancer			I.V. Oral	Reduced hyperalgesia
	Mostyn et al. (2019)	PSNL	Rats	30 mg/kg	I.P.	Reduced mechanical allodynia. Abdominal pain side effects.
	Mohammadzadeh et al. (2021)			4 mg/kg	S.C.	Reduced mechanical allodynia
Compound 1	Mingorance-Le Meur et al. (2013)	Formalin	Mice	25, 100 mg/kg	I.P.	Reduced mechanical allodynia
Oleoyl-D-Lysine	Mostyn et al. (2019)	PSNL	Rats	30 mg/kg	I.P.	Reduced mechanical allodynia
	Wilson et al. (in preparation)	CCI	Mice	1–100 mg/kg		
		CFA				No analgesia
		Hot plate				
Opiranserin	Pang et al. (2012)	SNL	Rats	25 mg/kg	S.C. Oral	Reduced mechanical allodynia
		Formalin			S.C.	Reduced pain-related behaviors
ORG25543 3-pyridyl amide derivative	Imam et al., (2020) Kuo et al., (2021)	PCIBP	Rats	10 mg/kg 3–30 mg/kg	Oral	Evoked partial pain relief
		CIPN				Reduced mechanical allodynia and hyperalgesia

4-(benzyloxy)-N-[1-(dimethylamino)cyclopentyl]methyl]-3,5-dimethoxybenzamide (ORG25543) is selective for GlyT2 over GlyT1 and binds irreversibly, producing dose-dependent reductions in allodynia and hyperalgesia following I.V. administration in mouse PSNL, bone cancer, and formalin pain models (Morita et al., 2008; Mingorance-Le Meur et al., 2013; Motoyama et al., 2014; Cioffi, 2021). However, ORG25543 causes tremors at low doses and seizures and/or death at higher doses when compared to GlyT2^−/−^ mice (Mingorance-Le Meur et al., 2013). This is likely due to the irreversible binding at GlyT2 depleting intracellular glycine and preventing glycinergic neurotransmission. A reversible analog to ORG25543 was developed by Mingorance-Le Meur et al. (2013) termed *Compound, 1* that proved analgesic in a mouse formalin model without causing tremors or convulsions. This supports the suggestion that reversible GlyT2 inhibitors are favorable over irreversible.

The endogenous acyl amino acid, N-arachidonoyl glycine (NaGly), inhibits GlyT2 and is found at its highest concentrations within the spinal cord (Huang et al., 2001; Wiles et al., 2006). A series of lipid derivatives of NaGly were developed by Mostyn et al. (2019), with one compound, oleoyl-D-lysine, showing greater anti-allodynia than ORG25543 in a rat PSNL model. Oleoyl-D-lysine also had a significantly milder side effect profile when compared to ORG25543.

VVZ-149 (opiranserin) is a structural analog of ORG25543, with dual antagonism at GlyT2 and 5-HT_2A_ receptors. In rat SNL and formalin models of pain, 25 mg/kg S.C. opiranserin effectively reduced mechanical allodynia and pain-related behaviors with efficacy comparable to 3 mg/kg morphine (Pang et al., 2012). Oral administration of opiranserin also reduced mechanical allodynia in a rat SNL model. It has been proposed that dual antagonism at GlyT2 and 5-HT_2A_ receptors provides effective analgesia through synergistic activity. Opiranserin has progressed through to phase III clinical trials, currently being the only GlyT2 inhibitor to reach this juncture. An orally available 3-pyridyl amide derivative of ORG25543 has shown promise in cancer models of pain. In a rat CIPN model, oral administration of up to 30 mg/kg of the inhibitor reduced mechanical allodynia and hyperalgesia (Kuo et al., 2021). Likewise, the inhibitor partially alleviated pain produced in a rat prostate cancer-induced bone pain (PCIBP) model (Imam et al., 2020).

### GlyR-Positive Allosteric Modulators

With access to higher quality receptor structures and ligand-binding sites, the development of positive allosteric modulators of GlyR has increased in recent years (Table 3). AM-1488 is a tricyclic sulphonamide which potentiates GlyRα1 and GlyRα3 (Bregman et al., 2017). In a mouse SNI model of pain, oral administration of AM-1488 reduced tactile allodynia with efficacy comparable to gabapentin.

**TABLE 3:** Experiments investigating known GlyR-positive allosteric modulators.

Compound	References	Model	Animal	Dose	ROA	End points
AM-1488	Bregman et al. (2017)	SNI	Mice	20 mg/kg	Oral	Reduced tactile allodynia
LT-01-25	Leuwer et al. (2017)	PSNL	Rats	10, 30 mg/kg	Oral	Reduced mechanical allodynia and cold hyperalgesia
		Diabetic neuropathy		≤100 mg/kg		Reduced mechanical allodynia
2,6-DTBP	Acuña et al. (2016)	Zymosan inflammatory	Mice	90 mg/kg	I.P.	Reduced mechanical allodynia and thermal hyperalgesia
		CFA GlyRα3^−/−^				Reduced mechanical allodynia in WT but not knockout animals
		CCI GlyRα3^−/−^				Reduced mechanical allodynia in both WT and knockout animals
DH-CBD	Xiong et al. (2012)	CFA	Rats	100 µg	I.T.	Dose-dependent reduction of mechanical and thermal allodynia
		SNL GlyRα3^−/−^				Suppressed mechanical allodynia in WT but not knockout mice
THC	Xiong et al. (2011)	CB1/2^−/−^ GlyRα3^−/−^	Mice	10 mg/kg	I.P.	Provided analgesia in acute tail-flick test in animals with CB knockout but not GlyRα3 knockout

6-Di-tert-butylphenol (2,6-DTBP) is a propofol derivative that potentiates GlyRα1 and GlyRα3 (Ahrens et al., 2004; Ahrens et al., 2009). Intraperitoneal injection of 2,6-DTBP significantly reduces mechanical allodynia and thermal hyperalgesia in a mouse Zymosan inflammatory pain model and reduces mechanical allodynia in mice CFA and CCI models of pain (Acuña et al., 2016). Acuña et al. (2016) further investigated 2,6-DTBP in GlyRα3^−/−^ mice, finding that the anti-allodynic effect in CFA, but not CCI mice, was lost. This is consistent with the role that GlyRα3 has in inflammatory pain states and suggests that restoration of normal pain states does not require α3 subunit-specific modulation. Another propofol derivative, LT-01-25, is a selective GlyRα1-positive allosteric modulator, currently under patent (Leuwer et al., 2017). Oral administration of LT-01-25 produced a near-complete reversal of mechanical allodynia in rat PSNL and diabetic neuropathy models, with no observed side effects.

Although α2-containing GlyR appear to be upregulated in animal models of neuropathic pain, due to subunit homogeneity, there are currently no modulators directly targeting these receptors. This may be an avenue to explore future drug development in the treatment of neuropathic pain.

Cannabinoids have been shown to have potentiating properties at GlyR. I.T. administration of dehydroxylcannabidiol (DH-CBD) dose-dependently reduced mechanical allodynia in wild-type rat CFA and CCI models of pain (Xiong et al., 2012). Notably, these analgesic effects were attenuated in GlyRα3^−/−^ rats (Xiong et al., 2012). Xiong et al. (2011) observed that I.P. injection of Δ9-tetrahydrocannabinol (THC) provided analgesia in an acute tail-flick reflex test in CB1^−/−^ and CB2^−/−^, but not GlyRα3^−/−^ mice. These findings suggest that α3 subunit-containing GlyR has a role in cannabinoid-mediated analgesia.

## Conclusion

The findings of this review demonstrate the value of animal models in both elucidating the mechanisms of neuropathic pain and providing the means to investigate potential therapies those restore normal pain signaling. While animal studies are not without limitations, the quality of information gained from behavioral studies is instrumental in drug development.
